# Pre-Eclampsia Biomarkers for Women With Type 1 Diabetes Mellitus: A Comprehensive Review of Recent Literature

**DOI:** 10.3389/fbioe.2022.809528

**Published:** 2022-05-26

**Authors:** Katrina Z. Freimane, Lauren Kerrigan, Kelly-Ann Eastwood, Chris J. Watson

**Affiliations:** ^1^ Wellcome-Wolfson Institute for Experimental Medicine, Queen’s University Belfast, Belfast, United Kingdom; ^2^ School of Medicine, Dentistry and Biomedical Sciences, Centre for Public Health, Queen’s University Belfast, Belfast, United Kingdom; ^3^ Department of Fetal Medicine, St. Michael’s Hospital, University Hospitals Bristol and Weston NHS Foundation Trust, Bristol, United Kingdom

**Keywords:** pre-eclampsia (PE), type 1 diabetes mellitus, biomarkers, pregnancy complications, pregestational diabetes, narrative review

## Abstract

**Background:** Pre-eclampsia is a serious consideration for women with type 1 diabetes mellitus (T1DM) planning pregnancy. Risk stratification strategies, such as biomarkers measured in the first trimester of pregnancy, could help identify high-risk women. The literature on T1DM-specific pre-eclampsia biomarkers is expanding. We aimed to provide a narrative review of recently published evidence to identify the most promising biomarker candidates that could be targeted for clinical implementation in existing PE models.

**Methods:** A search using MeSH terms was carried out of Medline, EMBASE, Maternity and Infant Care, Web of Science, and Scopus for relevant papers published since 2015 inclusive and in English. The time limit was applied from the publication of the preceding systematic review in this field. Included studies had pre-eclampsia as a primary outcome, measured one or more serum, plasma or urine biomarkers at any time during pregnancy, and had a distinct group of women with T1DM who developed pre-eclampsia. Studies with pre-eclampsia as a composite outcome were not considered. No restrictions on study types were applied. A narrative synthesis approach was adopted for analysis.

**Results:** A total of 510 records were screened yielding 18 eligible studies relating to 32 different biomarkers. Higher first-trimester levels of HbA1c and urinary albumin were associated with an increased risk of pre-eclampsia development in women with T1DM. Urinary neutrophil gelatinase-associated lipocalin and adipokines were novel biomarkers showing moderate predictive ability before 15 gestational weeks. Two T1DM-specific pre-eclampsia prediction models were proposed, measuring adipokines or urinary neutrophil gelatinase-associated lipocalin together with easily attainable maternal clinical characteristics. Contradicting previous literature, pre-eclampsia risk in women with T1DM was correlated with vitamin D levels and atherogenic lipid profile in the context of haptoglobin phenotype 2-2. Pregnancy-associated plasma protein-A and soluble endoglin did not predict pre-eclampsia in women with T1DM, and soluble Fms-like tyrosine kinase 1 only predicted pre-eclampsia from the third trimester.

**Conclusion:** Maternally derived biomarkers reflecting glycemic control, insulin resistance and renal dysfunction performed better as PE predictors among women with T1DM than those derived from the placenta. These biomarkers could be trialed in current PE prediction algorithms to tailor them for women with T1DM.

## Introduction

Pre-eclampsia (PE) is a hypertensive multisystem disorder of pregnancy that has been subject to intense research scrutiny. Its pathophysiology and factors dictating maternal susceptibility remain incompletely understood. The mortality and morbidity burden of PE is high, with women in low- and middle-income countries disproportionately affected (Say et al., 2014). Delivery remains the only definitive cure presenting a clinical dilemma where fetal maturity is balanced against maternal risks of continuing the pregnancy (National Institute for Health and Care Excellence (NICE), 2019). Women with pregestational type 1 diabetes mellitus (T1DM) have a five-fold risk of PE compared to the general population (Weissgerber and Mudd, 2015), with the risk even higher with pre-existing diabetic microvascular disease (Leguizamón et al., 2015).

Management of pregnancy with T1DM is challenging and resource-intensive. Current practice includes the initiation of aspirin prophylaxis from 12 weeks' gestation (National Institute for Health and Care Excellence (NICE), 2019; American College of Obstetrics and Gynecology (ACOG), 2018), although some cohorts of high-risk women have been resistant to aspirin therapy (Caritis et al., 1998; Villa et al., 2013; Rolnik et al., 2017a). No clinical trials have been carried out randomizing women with T1DM to aspirin or placebo prior to 12 weeks' gestation with an ongoing phase III randomized controlled trial by Finnegan et al. (2019) using placental dysfunction as a composite outcome. Prediction and risk stratification remain research priorities in this high-risk group and preventatives other than aspirin are yet to be found. Alleviation of the highly medicalized pregnancy course for women with T1DM is vital to improve patient satisfaction and optimize the use of healthcare resources, considerations relevant to an increasingly overwhelmed health service. A biomarker for PE specific to pregnancies complicated by T1DM has the potential to address this need.

Women with T1DM have been historically underrepresented in PE research (Weissgerber and Mudd, 2015), resulting in an unclear understanding of what accounts for the exaggerated PE risk in this group. No major advances have been made in this field since White’s classification (White, 1949) and generalizing findings from studies of existing PE prediction models, such as the Fetal Medicine Foundation (FMF) algorithm, is problematic due to the small number of women with T1DM in the study population (Rolnik et al., 2017b). This is despite previous reports of PE biomarkers performing differently in pregnancies complicated by pregestational diabetes (Zen et al., 2019). Moreover, large clinical trials investigating PE biomarkers and risk prediction models often pool data from type 1 and type 2 diabetes mellitus groups together or exclude them completely (Agrawal et al., 2017; Guy et al., 2020; Serra et al., 2020). As a result, an important research gap remains in the development of predictive modalities for PE in women with T1DM. To date, only one comprehensive systematic review of T1DM-specific PE biomarkers has been carried out by Wotherspoon et al. (2016a), which did not recommend any individual biomarker for clinical implementation. We aim to provide a comprehensive review of recently published evidence of biomarker candidates for PE among women with T1DM that could be targeted for clinical implementation in existing PE prediction models.

## Methods

### Electronic Database Search

A search was carried out using the electronic databases Medline, EMBASE, Maternity and Infant Care, Web of Science, and Scopus on 11 July 2020 (Table 1). Searches were limited to human studies published since 2015 inclusive to identify all relevant publications after the search carried out by Wotherspoon et al. on 16 January 2015 (Wotherspoon et al., 2016a). No limitations on study type were applied. All the articles included were available in English. The database-specific formatting of keywords was combined with the use of medical subject headings to maximize the number of results. Once all duplicates had been eliminated, articles were screened by title and abstract to identify those relevant for PE prediction in women with T1DM. Full-text manuscripts were obtained for the selected articles and assessed for inclusion in the review. To identify any omitted articles in the search, reference lists of the included articles were scanned and a broader search was carried out on Medline using only search terms relating to PE and T1DM (Table 1). Inclusion and exclusion criteria applied are outlined in Supplementary Appendix S1.

**TABLE 1:** Syntax used for searching electronic databases.

1.	Preeclampsia OR pre-eclampsia OR pregnancy-induced hypertension OR pregnancy induced hypertension OR hypertensive disorder of pregnancy OR toxaemia of pregnancy OR toxemia of pregnancy OR gestosis
2.	Pregnancy in diabetics OR pregravid diabetes OR pre-pregnancy diabetes OR pregestational diabetes OR diabetes mellitus OR type 1 diabetes OR type 1 diabetes mellitus OR type one diabetes OR type one diabetes mellitus OR insulin dependent diabetes mellitus OR IDDM OR T1DM OR juvenile diabetes OR juvenile-onset diabetes
3.	Biomarker OR biological marker OR biochemical marker OR molecular marker
4.	#1 AND #2 AND #3

### Data Extraction

A data extraction form was used to record key information about the design of each study including study type, cohort country of origin, number of women with T1DM, diabetes duration, age distribution, the prevalence of PE within the cohort and ethnicity distribution. For biomarker data, information was extracted using a data extraction form including the biomarkers used, measurement timeframe within gestation, main findings and measures of predictive potential used.

### Data Analysis

Due to the heterogeneity of biomarkers used within the included studies, a meta-analysis could not be carried out and a narrative synthesis approach was adopted instead. An assessment of overlapping data between studies included in this review, and Wotherspoon et al. (2016a) can be found in Supplementary Appendix S2.

## Results

### Study Selection

**FIGURE 1:**
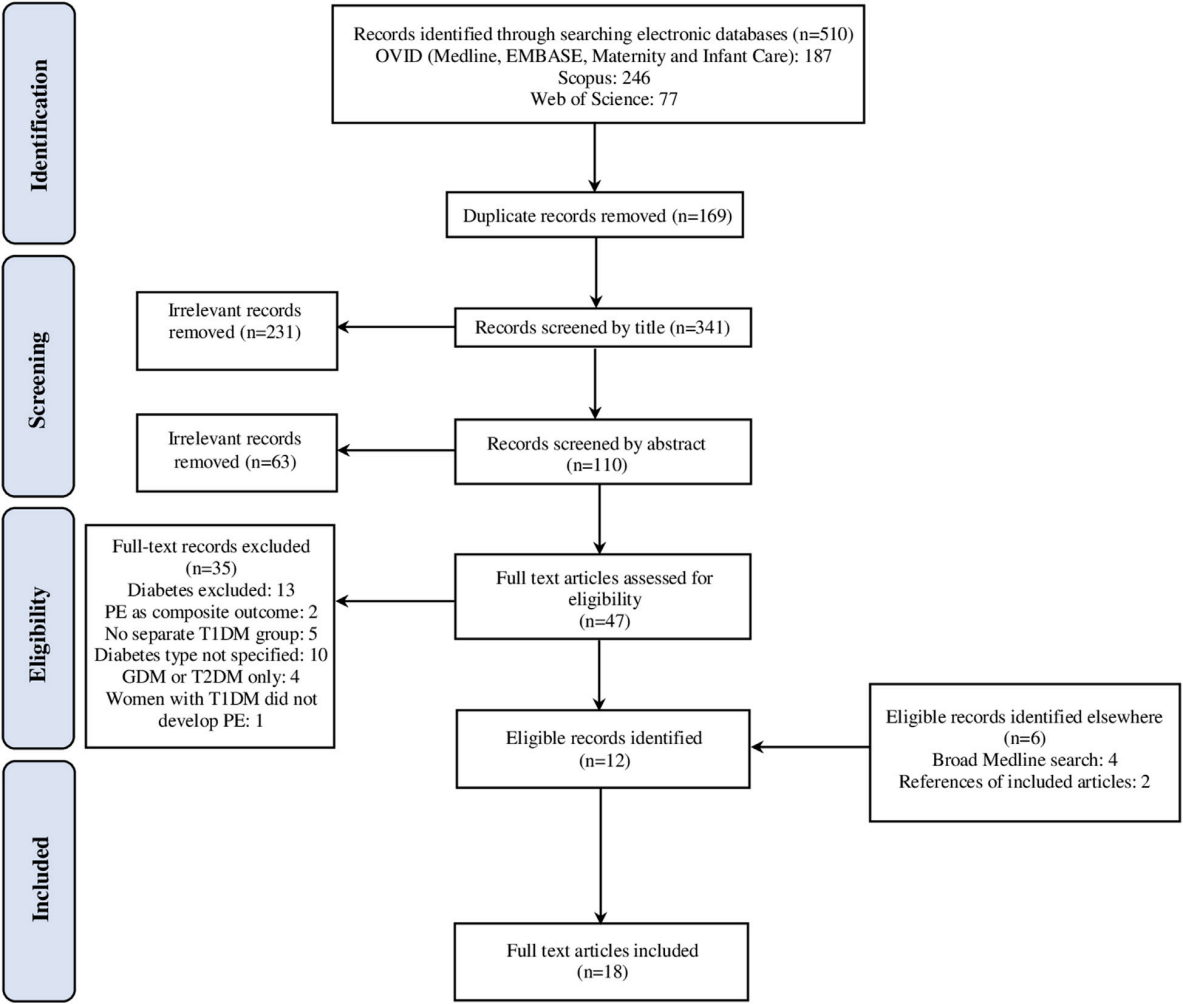
The Preferred Reporting Items for Systematic Reviews and Meta-Analyses (PRISMA) flow diagram of the study selection process.

A total of 510 records were identified which was reduced to 341 after the removal of duplicates and 47 after screening by title and abstract. Full-text articles were acquired for these and subsequently, 35 publications were excluded, with 18 remaining studies selected for inclusion in the final review. The process of article selection is detailed in a PRISMA diagram in Figure 1

### Characteristics of Included Studies

A detailed summary of study and cohort characteristics can be found in Table 2. Of the 18 studies selected for the current review, three were systematic reviews (Vestgaard et al., 2017a; Cavero-Redondo et al., 2018; Xiang et al., 2018), six were case-control studies (Basu et al., 2015; Tsiakkas et al., 2015; Gutaj et al., 2017; Kelly et al., 2017; Kelly et al., 2018; Kelly et al., 2020) and nine were cohort studies (Maresh et al., 2015; Wotherspoon et al., 2016b; Klemetti et al., 2016; Lauszus and Fuglsang, 2016; Vestgaard et al., 2017b; Nielsen et al., 2018; Kelly et al., 2019; Perea et al., 2019; Kapustin et al., 2020). The number of women with T1DM in the cohorts ranged from 47 to 1,094, with Vestgaard et al. and Xiang et al. having pooled 11,518 and 3,239 participants with T1DM, respectively (Vestgaard et al., 2017a; Xiang et al., 2018). A total of 27 cohorts represented 11 European countries and Australia, the United States, Russia and Brazil. PE prevalence within cohorts ranged from 4.8% to 35.9%. There was heterogeneity in the timeframe of biomarker measurements, with 12 studies (Basu et al., 2015; Tsiakkas et al., 2015; Klemetti et al., 2016; Lauszus and Fuglsang, 2016; Gutaj et al., 2017; Kelly et al., 2017; Cavero-Redondo et al., 2018; Kelly et al., 2018; Nielsen et al., 2018; Kelly et al., 2019; Perea et al., 2019; Kelly et al., 2020) recording biomarker levels across all three trimesters of pregnancy, four studies (Maresh et al., 2015; Wotherspoon et al., 2016b; Vestgaard et al., 2017a; Vestgaard et al., 2017b) in any two trimesters and one study (Kapustin et al., 2020) taking measurements in the first trimester only. The timing of biomarker measurements was not specified in Xiang et al. (2018). Of the 32 different biomarkers identified, glycosylated hemoglobin, serum lipid-associated molecules and urinary protein were most commonly discussed with a smaller number of studies investigating adipokines or angiogenic factors. Several novel markers were described including vitamin D and urinary neutrophil gelatinase-associated lipocalin. A narrative synthesis of the main results of included studies is contained in Table 3 **.**

**TABLE 2:** Study and population characteristics.

Author, year	Women with T1DM (n)	PE prevalence in T1DM, n (%)^a^	Duration of T1DM (years)^b^	Age distribution (years)^c^	Ethnicity	Country
Basu et al. (2015)	47	24 (35.9%)	T1DM + PE+: 16.8 ± 6.8	T1DM + PE+: 28.5 ± 5.6	86% Caucasian	Australia, United States, Norway^d^
			T1DM + PE−: 14.8 ± 7.0	T1DM + PE−: 29.9 ± 3.8		
Cavero-Redondo et al. (2018)	2559 (pooled)	11.0–28.6%	8.4–15.4	23.5–29.7	Not recorded	4 studies pertaining to 5 cohorts originating from Finland, the United Kingdom, Denmark, and Bulgaria
Gutaj et al. (2017)	165	16 (9.7%)	Control: 12 ± 7	Control: 29 ± 4	Caucasian	Poland
			Gestational HTN: 12 ± 8	Gestational HTN: 29 ± 7		
			PE+: 17 ± 7	PE+: 27 ± 4		
Kapustin et al. (2020)	100	25 (25%)	Not recorded	27.2–30.1	Not recorded	Russia
Kelly et al. (2017)	47	24 (35.9%)	T1DM + PE+: 16.8 ± 6.8	T1DM + PE+: 28.5 ± 5.6	86% Caucasian	Australia, the United States, and Norway^d^
			T1DM + PE−: 14.8 ± 7.0	T1DM + PE−: 29.9 ± 3.8		
Kelly et al. (2018)	47	23 (35.9%)	T1DM + PE+: 16.8 ± 6.8	T1DM + PE+: 28.5 ± 5.6	86% Caucasian	Australia, the United States, and Norway^d^
			T1DM + PE−: 14.8 ± 7.0	T1DM + PE−: 29.9 ± 3.8		
Kelly et al. (2019)	47	23 (35.9%)	T1DM + PE+: 16.8 ± 6.8	T1DM + PE+: 28.5 ± 5.6	86% Caucasian	Australia, the United States, and Norway^d^
			T1DM + PE−: 14.8 ± 7.0	T1DM + PE−: 29.9 ± 3.8		
Kelly et al. (2020)	47	24 (35.9%)	T1DM + PE+: 16.8 ± 6.8	T1DM + PE+: 28.5 ± 5.6	86% Caucasian	Australia, the United States, and Norway^d^
			T1DM + PE−: 14.8 ± 7.0	T1DM + PE−: 29.9 ± 3.8		
Klemetti et al. (2016)	1094	260 (23.7%)	White B: 4 (0–9)	White B: 31.5 ± 4.4	Not recorded	Finland
			White C: 13 (2–19)	White C: 29.7 ± 5.4		
			White D: 22 (6–36)	White D: 30.8 ± 5.2		
			White R: 24 (11–36)	White R: 31.6 ± 4.4		
			White F: 20 (10–34)	White F: 30.0 ± 4.9		
Lauszus and Fuglsang (2016)	97	32 (33%)	Normoalbuminuric: 12 ± 8	Normoalbuminuric: 28 ± 4	Not recorded	Denmark
			Microalbuminuric: 14 ± 8	Microalbuminuric: 27 ± 5		
			Macroalbuminuric: 20 ± 5	Macroalbuminuric: 30 ± 4		
Maresh et al. (2015)	725	120 (17%)	14.5 ± 8.2	29·5 ± 5.6	96.5% Caucasian	United Kingdom^e^
					0.5% Black	
					1.5% Asian	
					1.5% Other/not known	
Nielsen et al. (2018)	88	14 (16%)	T1DM + PE+: 16.2 ± 7.5	T1DM + PE+: 29.5 ± 5.9	Caucasian	Denmark
			T1DM + PE−: 13.3 ± 8.9	T1DM + PE−: 29.9 ± 4.5		
Perea et al. (2019)	77	14 (18%)	IAH: 19.4 ± 8.6	IAH: 33.7 ± 3.2	Caucasian	Spain
			NAH: 16.1 ± 8.6	NAH: 34.4 ± 4.0		
Tsiakkas et al. (2015)	228	11 (4.8%)	Not recorded	11 + 0 to 13 + 6 w: 31.1 (26.6–34.9)	72.5–73.1 Caucasian	United Kingdom
				19 + 0 to 24 + 6 w: 31.1 (26.6–34.8)	17.7–19.2% Afro-Caribbean	
				30 + 0 to 37 + 6 w: 31.1 (26.7–34.8)	3.7–4.5% South Asian	
					2.0–2.3% East Asian	
					2.2–2.5% Mixed	
Vestgaard et al. (2017b)	198	16 (8%)	Preterm delivery: 15.0 (0.5–32.0)	Preterm delivery: 31 (19–39)	Caucasian	2 cohorts from Denmark
			Term delivery: 15.0 (0.5–38.0)	Term delivery: 30 (21–43)		
Vestgaard et al. (2017a)	11,518 (pooled)	9.3–33.5%	Not recorded	Not recorded	Not recorded	11 studies pertaining to 11 cohorts from Italy, Denmark, United Kingdom, Sweden, Finland, the United States, and United Kingdom
Wotherspoon et al. (2016b)	710	120 (17%)	14.5 ± 8.2	29·5 ± 5.6	96.5% Caucasian	United Kingdom^e^
					0.5% Black	
					1.5% Asian	
					1.5% Other/not known	
Xiang et al. (2018)	3239 (pooled)	8.5–30.8%	Not recorded	25–40	Not recorded	11 studies pertaining to 11 cohorts from Poland, Slovakia, Brazil, Finland, Italy, Denmark, the United States of America, United Kingdom, and Sweden

HTN, hypertension; IAH, impaired awareness of hypoglycemia; NAH, normal awareness of hypoglycemia; T1DM + PE+, women with T1DM who developed pre-eclampsia; T1DM + PE−, women with T1DM who did not develop pre-eclampsia; w, weeks; y, years.

a PE prevalence within the T1DM, group of the cohort, given as a range for systematic reviews.

b Data given as mean ± SD, range or median (range).

c Data given as mean ± SD, mean, range, or mean (interquartile range).

d Studies by Basu et al. (2015), Kelly et al. (2017), Tsiakkas et al. (2015), Kelly et al. (2019), and Kelly et al. (2020) were based on participants from the same cohort.

e Studies by Maresh et al. (2015) and Wotherspoon et al. (2016b) were based on participants from the same cohort.

**TABLE 3:** Narrative synthesis of biomarkers for pre-eclampsia prediction in women with pregestational T1DM.

Study	Biomarkers	Measurements (w or w + days)^a^	Findings (T1DM + PE + vs. T1DM + PE-)	Predictive potential measure
Basu et al. (2015)	Copper, iron, manganese, selenium, zinc, HDL-c, LDL-c, triglycerides, and total cholesterol	V1: 12.3 ± 1.9 w	↑Zinc in T1	—
		V2: 21.6 ± 1.5 w	↑Zinc:HDL in T1	
		V3: 31.5 ± 1.7 w	↑Copper:zinc and ↑Copper:HDL-c throughout gestation	
			↑HDL-c at baseline	
Cavero-Redondo et al. (2018)	HbA1c	V1: T1 (7–12 w)	↑HbA1c at all visits	For 1% HbA1c ↑
		V2: T2/T3 (22–36 w)		OR = 1.37 in T1
				OR = 1.67 in T2/T3
Gutaj et al. (2017)	HbA1c and triglycerides	V1: <12 w	↑HbA1c at all visits	For ↑HbA1c
	HDL-c	V2: 20–24 w	↑Triglycerides in T3	OR = 1.38 in T1
	LDL-c	V3: 34–39 w		OR = 2.76 in T2
	Total cholesterol			OR = 2.42 in T3
				For ↑triglycerides
				OR = 5.32 in T1
				OR = 2.52 T2
				OR = 2.28 in T3
Kapustin et al. (2020)	PAPP-A, β-hCG	11 + 0 w to 13+6 w	NSD	—
Kelly et al. (2017)	Leptin, adiponectin (total, HMW), FABP4, resistin, and RBP4	V1: 12.3 ± 1.9 w	↑Leptin:total adiponectin at V1 and V2	Best prediction models using doubling of serum levels
		V2: 21.6 ± 1.5 w	↑Leptin:HMW adiponectin at V1 and V2	OR = 9.0, sensitivity 81%, specificity 80%, PPV 100%, NPV 69% for leptin in T1
		V3: 31.5 ± 1.7 w	↑FABP4 at V2 and V3	OR 3.7, sensitivity 84%, specificity 68%, PPV 100%, NPV 56% for leptin:total adiponectin in T2
			↑Leptin, ↑FABP4:total adiponectin, and ↑FABP4:HMW adiponectin throughout pregnancy	OR 25.1, sensitivity 71%, specificity 75%, PPV 100%, NPV 65% for FABP4 in T3
			↓Total adiponectin at V1	
			↓HMW adiponectin at V1 and V2	
Kelly et al. (2018)	uNGALcc, pNGAL, creatinine, and KIM-1	V1: 12.3 ± 1.9 w	↑uNGALcc at V1	↑uNGALcc (leukocyte-negative): sensitivity 75%, specificity 70%, PPV 32%, NPV 93% before 15 w
		V2: 21.6 ± 1.5 w	NSD in pNGAL	
		V3: 31.5 ± 1.7 w	NSD in urinary KIM-1	
			↑eGFR at V1 but NSD after adjustment	
Kelly et al. (2019)	Hp, LDL-c, LDL particle concentration, ApoB, ApoA1, triglyceride:HDL-c, ApoB:ApoA1, sFlt-1, sEng, PlGF, and sFlt-1/PlGF	V1: 12.3 ± 1.9 w	↑sFlt-1, sFlt-1/PlGF at V3	—
		V2: 21.6 ± 1.5 w	↓PlGF at V3	
		V3: 31.5 ± 1.7 w	Within the Hp 2–2 only	
			↑LDL-c at V1 and V2	
			↑LDL particle concentration, ↑ApoB, ↑Triglyceride:HDL-c, and ↑ApoB/ApoA1 at all visits	
			↓HDL-c, ApoA1, and large HDL particle concentrations at all visits	
Kelly et al. (2020)	25(OH)D 1	V1: 12.3 ± 1.9 w	↑1,25(OH)_2_D at V2 (free, total, and bioavailable) and V3 (bioavailable and free)	For every 1 pg/ml ↑
	25(OH)_2_D	V2: 21.6 ± 1.5 w	↑1,25(OH)_2_D/25(OH)D at V3	OR = 1.28 for 1,25(OH)2D at T2
	VDBP	V3: 31.5 ± 1.7 w	↑1,25(OH)_2_D/VDBP at V2 and V3	OR = 1.18 for 1,25(OH)_2_D at T3
			↓VDBP at V3	For every unit ↑
				OR = 2.71 for 1,25(OH)_2_D/VDBP at T2
				OR = 2.53 for 1,25(OH)_2_D/VDBP at T3
Klemetti et al.(2016)	Macroalbuminuria, HbA1c	HbA1c	Stepwise ↑ in White’s class from B to F corresponded to ↑PE incidence HbA1c ≥ 7% in T1	For HbA1c ≥ 7%: OR = 1.92 in T1
		V1: last value within 12 months before pregnancy; V2: first value in T1; V3: 18+0 w to 22+0 w; V4: last value before delivery		For macroalbuminuria: OR = 8.65 in T1
		Macroalbuminuria		
		V1: 6–10 w; V2: 11–13 w; every 2-4 w during T2; every 1-2w during T3		
Lauszus and Fuglsang (2016)	IGF-1	V1: 14 w, V2: 18 w, V3: 22 w, V4: 26 w, V5: 30 w, V6: 32 w	NSD	—
Maresh et al. (2015)	HbA1c	V1: 26 w	↑HbA1c at all visits	Compared to HbA1c <6.0%
		V2: 34 w		OR = 4.3 for 6.5–6.9%, OR = 4.6 for 7.0–7.4%, OR = 5.1 for ≥7.5% at 26 w
				OR = 3.0 for 6.5–6.9%, OR = 5.3 for 7.0–7.4%, OR = 6.8 for ≥7.5% at 34 w
Nielsen et al. (2018)	Urine PCR	V1: 12 w, V2: 20 w, V3: 28 w, V4: 32 w, V5: 36 w	↑Urine PCR at V5	For ↑Urine ACR
	Plasma aldosterone		↑Plasma aldosterone at V2	OR = 3.92 at 12 w, OR = 3.43 at 20 w, OR = 3.58 at 28 w, OR = 6.15 at 32 w, OR = 17.94 at 36 w, AUC = 0.91 at 36 w
	Urine ACR		↑Urine ACR at all visits	For ↑Urine PCR
	HbA1c		↑HbA1c from V2 onwards	OR = 2.49 and AUC = 0.74 at 36 w
				For each 1% HbA1c↑
				OR = 1.08 at 20 w, OR = 1.09 at 28 w, OR = 6.15 at 32 w, OR = 17.94 at 36 w
Perea et al. (2019)	Total cholesterol Triglycerides HDL-c	V1: 8–14 w	↑Triglycerides at V2	For each 10 mg/dl ↑Triglycerides: OR = 1.32 - -
		V2: 22–28 w		
		V3: 31–36 w		
Tsiakkas et al. (2015)	MoMs of serum PlGF	V1: 11 + 0 to 13 + 6	↓PlGF MoM	—
		V2: 19 + 0 to 24 + 6 w		
		V3: 30 + 0 to 34 + 6 w/35 + 0 to 37 + 6 w		
Vestgaard et al. (2017b)	25(OH)D	V1: 8 (5–14 w)	Non-significantly ↓25(OH)D at baseline	
		V2: 24 (32–36 w)		
Vestgaard et al. (2017a)	Microalbuminuria, macroalbuminuria, and HbA1c	<16–20 w	Presence of microalbuminuria, macroalbuminuria, and ↑HbA1c from T1	For microalbuminuria: OR = 3.8 and OR = 11.7 in T1
				For macroalbuminuria: OR = 4.7–23.5 in T1
Wotherspoon et al. (2016b)	FABP4	V1: 14 w (8–22 w)	↑FABP4 at V1 and V2	For each doubling of FABP4: OR = 1.4 at V1, OR = 1.6 at V2
		V2: 26 w	↑FABP4 at 13 w	
Xiang et al. (2018)	Microalbuminuria and macroalbuminuria	Not recorded	Presence of microalbuminuria and macroalbuminuria	For microalbuminuria: OR = 4.19
				For macroalbuminuria: OR = 7.19

ApoA1, apolipoprotein A1; ApoB, apolipoprotein B; HMW, high molecular weight; IGF-1, insulin-like growth factor 1; KIM-1, urinary kidney injury molecule-1; NSD, no significant difference; RBP4, retinol-binding protein 4; T1–T3, trimesters 1 to 3; V1–V6, visits 1 to 6; w, weeks.

a Data given as mean ± SD, mean, or median (range).

### Glycosylated Hemoglobin

The predictive potential of plasma glycated hemoglobin A1c (HbA1c) was examined in six studies (Maresh et al., 2015; Klemetti et al., 2016; Vestgaard et al., 2017a; Gutaj et al., 2017; Cavero-Redondo et al., 2018; Nielsen et al., 2018). PE risk was correlated to HbA1c ≥ 7% and >6% (Klemetti et al., 2016; Gutaj et al., 2017) cut-offs denoting “poor” glycemic control or 0.5% and 1% HbA1c increments (Maresh et al., 2015; Cavero-Redondo et al., 2018; Nielsen et al., 2018). With the exception of two studies (Hsu et al., 1996; Ekbom et al., 2001) included by Vestgaard et al. (2017a) in their systematic review, a positive association between rising HbA1c and PE was seen, although the odds ratios (OR) differed between studies and trimesters. For HbA1c cut-offs indicating poor glycemic control, ORs were 1.38–1.92 in the first trimester, 2.76 in the second trimester, and 2.42 in the third trimester (Klemetti et al., 2016; Gutaj et al., 2017). Comparatively, every 1% HbA1c increase produced OR = 1.37 in the first trimester, OR = 1.08–1.67 in the second trimester, and OR = 6.15–17.94 in the third trimester (Cavero-Redondo et al., 2018; Nielsen et al., 2018). In the second trimester, OR was 4.3 for HbA1c 6.5%–6.9% compared to OR of 4.6 for HbA1c 7.0–7.4%, and in the third trimester OR was 3.0 for HbA1c 6.5%–6.9% compared to OR of 5.3 for HbA1c 7.0%–7.4% and OR of 6.8 for HbA1c ≥ 7% (Maresh et al., 2015).

### Urinary Protein

Five studies investigated some form of urinary protein (Klemetti et al., 2016; Vestgaard et al., 2017a; Kelly et al., 2018; Nielsen et al., 2018; Xiang et al., 2018). A strong association was shown between urinary albumin excretion and PE development, with a higher risk demonstrated for women with macroalbuminuria rather than microalbuminuria. The presence of microalbuminuria prior to 16–20 weeks of gestation was associated with OR = 3.8 (Castiglioni et al., 2014) and 11.7 (Ekbom et al., 2001) for subsequent PE diagnosis in Vestgaard et al. (2017a). A pooled OR of 4.19 for microalbuminuria was calculated by Xiang et al. (2018), although the gestational age to which this applied was not specified. For macroalbuminuria, the pooled first trimester ORs were 7.19 (Klemetti et al., 2016) and 8.65 (Vestgaard et al., 2017a; Nielsen et al., 2018) which contrasted with the wide range of ORs (4.7–23.5) collated in Vestgaard et al. (2017a). Nielsen et al. (2018) investigated urine plasminogen/creatinine ratio (PCR) and urine albumin/creatinine ratio (ACR). The authors demonstrated an association between increased ACR and PE risk across the whole gestation, with ORs = 3.92 at 12 weeks and 3.43 at 20 weeks. However, a significant area under the curve (AUC) improvement for PE prediction using ACR compared to clinical variables was only found at 36 weeks. Urine PCR performed poorly, only showing an association with PE at 36 weeks.

A novel marker of renal cell injury, urinary neutrophil gelatinase-associated lipocalin (uNGALcc), was found to accurately predict PE from late first trimester (Kelly et al., 2018). Significantly raised uNGALcc was sustained in leukocyte-negative samples of women with subsequent PE diagnosis, which was important as NGAL is produced by activated neutrophils as well as damaged renal epithelium (Kelly et al., 2018). A model incorporating uNGALcc together with body mass index, HbA1c, and daily insulin dose had 75% sensitivity and 70% specificity for PE prediction prior to 15 weeks with a non-significant AUC improvement.

### Lipid-Associated Molecules

Six studies examined circulating cholesterol, triglycerides, adipokines and lipoproteins (Basu et al., 2015; Wotherspoon et al., 2016b; Gutaj et al., 2017; Kelly et al., 2017; Kelly et al., 2019; Perea et al., 2019). Data regarding levels of high-density lipoprotein cholesterol (HDL-c), low-density lipoprotein cholesterol (LDL-c), total cholesterol and PE development were conflicting. Two studies (Gutaj et al., 2017; Perea et al., 2019) found no difference in the levels of either molecule between PE and normotensive groups, contrasting with Basu et al. (2015) who showed a significant first-trimester HDL-c fall in PE. Kelly et al. (2019) examined whether an atherogenic lipid profile in PE is confined to certain haptoglobin phenotypes (Hp) and found that increased LDL-c and decreased HDL-c across the whole gestation existed only in women with T1DM who had Hp 2-2. The triglyceride data were also inconsistent. Contrasting with Basu et al. (2015) who found no difference in triglyceride levels between women who developed PE and those who did not others demonstrated higher triglyceride levels in women who developed PE across all three trimesters (Gutaj et al., 2017; Perea et al., 2019). Kelly et al. (2019) did not find increased triglyceride levels in women with PE with Hp 2-2 although they did show an increased triglyceride:HDL-c ratio across the whole gestation. Additionally, Kelly et al. (2019) found significantly upregulated ApoA1 and ApoB:ApoA1 throughout pregnancy in women with the Hp2-2 phenotype who developed PE.

Two studies examined adipokines (Wotherspoon et al., 2016b; Kelly et al., 2017). Fatty acid-binding protein 4 (FABP4) was elevated across all trimesters with OR = 1.4 at 14 weeks, 1.6 at 26 weeks (Wotherspoon et al., 2016b) and 25.1 at 31.5 weeks (Kelly et al., 2017) respective to each doubling of serum FABP4. In addition, Kelly et al. (2017) used HbA1c, daily insulin dose and gestational age to develop trimester-specific PE prediction models incorporating adipokines. In a third-trimester model, FABP4 was 71% sensitive and 75% specific for subsequent PE development. In the same study leptin was upregulated throughout pregnancy in women subsequently diagnosed with PE, and total and high molecular weight forms of adiponectin were decreased in the first and second trimesters. A first-trimester prediction model using doubling of serum leptin had OR = 9.0, with 81% sensitivity and 80% specificity, and a second-trimester prediction model using doubling of serum leptin:total adiponectin ratio had OR = 3.7, with 84% sensitivity and 68% specificity. The AUC improvements were non-significant for all prediction models using adipokines (Kelly et al., 2017).

### Other Plasma or Serum Biomarkers

Nine studies (Basu et al., 2015; Tsiakkas et al., 2015; Lauszus and Fuglsang, 2016; Vestgaard et al., 2017b; Kelly et al., 2018; Nielsen et al., 2018; Kelly et al., 2019; Kapustin et al., 2020; Kelly et al., 2020) examined other circulating molecules. Two studies (Tsiakkas et al., 2015; Kelly et al., 2019) investigated angiogenic factors soluble Fms-like tyrosine kinase 1 (sFlt-1), soluble endoglin (sEng) and placental growth factor (PlGF) in PE prediction among women with T1DM. PlGF was lower among women with T1DM developing PE (Tsiakkas et al., 2015) compared to normotensive women with T1DM but sEng was no different due to being consistently elevated in both groups. In the same study, sFlt-1, PlGF and sFlt-1/PlGF ratios were only predictive from the third trimester (Kelly et al., 2019). PAPP-A was found to be not different between groups of women with T1DM who developed PE or remained normotensive (Kapustin et al., 2020).

Two studies (Vestgaard et al., 2017b; Kelly et al., 2020) measured vitamin D in pregnant women with T1DM. Vitamin D deficiency was more common in T1DM groups in both studies but the predictive potential of vitamin D differed. The ORs were non-significant for the active form of vitamin D, 25(OH)D, in Vestgaard et al. (2017b) similar to Kapustin et al. (2020) However, Kapustin et al. (2020) were able to show significantly elevated levels of the precursor form of vitamin D, 1,25(OH)_2_D, and its ratio with vitamin D binding protein (VDBP) in second and third trimesters in PE groups. Every unit increase in 1,25(OH)_2_D:VDBP was correlated with OR = 2.71 in the second trimester and OR = 2.53 in the third trimester for later PE development.

## Discussion

Thirty-two different biomolecules spanning 5.5 years of published data were identified in this narrative review of PE biomarkers for women with T1DM. This compares to a similar number of biomolecules reported in Wotherspoon et al. (2016a), however, the previous review covered 25 years of literature, suggesting an expansion of the research field. Previously, Wotherspoon et al. (2016a) opted not to nominate any single PE biomarker for clinical implementation indicating a need for further validation of existing data and for discovery of novel candidates. The combined body of evidence between this review and Wotherspoon et al. (2016a) suggests that maternally derived biomarkers, such as HbA1c, urinary albumin and adipokines, are the highest performing predictors of PE among women with T1DM, with placental biomarkers such as PAPP-A showing less capacity to predict PE development in this population. These findings are in line with the growing body of evidence that some PE phenotypes are caused primarily by pre-existing cardiovascular compromise rather than placental dysfunction (Thilaganathan and Kalafat, 2019), with diabetes being one of the major known cardiovascular risk factors. Modern PE screening approaches, such as the FMF (Poon et al., 2009), incorporate biophysical, biochemical and ultrasonographic maternal parameters. Although evaluating the application of each parameter to women with T1DM is beyond the scope of this review, we propose that our findings are used to focus the search for alternatives to the biochemical components of these models, such as PAPP-A. Using biomarkers with known predictive performance and pathophysiological basis of action in PE development within a T1DM context would allow tailoring of such models to this high-risk population.

### Maternally Derived Biomarkers

The recent meta-analyses of HbA1c (Cavero-Redondo et al., 2018) and urinary albumin (Xiang et al., 2018) have validated previous observations (Wotherspoon et al., 2016a) in support of these biomarkers as predictors of PE among women with T1DM. Furthermore, HbA1c and urinary albumin are molecules whose upregulation in a high-risk PE state would be plausible, as they both directly relate to the current theories of the disease pathogenesis in this group. Hyperglycemia has a profoundly toxic effect on vascular function (Brownlee, 2001) and trophoblast viability (Inadera et al., 2010), with HbA1c reflecting a woman’s glycemic control over 6–8 weeks (Inkster et al., 2006). Comparatively, kidney damage detectable as albuminuria is a diagnostic feature of PE in both women with diabetes and the general population (National Institute for Health and Care Excellence (NICE), 2019). In women with T1DM, the placental pathology in PE stresses the kidneys whose functional reserve might have already been reduced by diabetic kidney disease, with albumin sometimes detectable in their urine even before pregnancy (Azzoug and Chentli, 2016; Mathiesen, 2016). Therefore, it is not surprising that the level of microalbuminuria, macroalbuminuria and HbA1c would be correlated with PE risk among women with T1DM (Leguizamón et al., 2015; Cavero-Redondo et al., 2018; Xiang et al., 2018).

Among studies within this review, HbA1c was used both as an independent biomarker and as part of prediction models for PE. Bearing in mind the physiological caveat of falling levels as pregnancy progresses, which has precluded HbA1c use beyond the first trimester in current practice (American College of Obstetrics and Gynaecology (ACOG), 2018; National Institute for Health and Care Excellence (NICE), 2015), HbA1c might be better placed for use in support of other PE biomarkers not affected by such physiological flux. However, validation of the HbA1c and PE relationship in a meta-analysis and the characterization of this using HbA1c increments beyond the arbitrary “poor” or “good” glycemic control thresholds (Maresh et al., 2015; Cavero-Redondo et al., 2018) indicate that its full clinical potential in pregnancy complicated by T1DM might not be realised. The clinical applications of HbA1c measurements during pregnancy might even extend beyond PE prediction to stratify the long-term risk of microangiopathy after PE development, an association already shown in non-pregnant women with diabetes (Gorst et al., 2015). Considering that the increased risk of diabetic retinopathy (Lövestam-Adrian et al., 1997; Gordin et al., 2013) and nephropathy (Gordin et al., 2007) is well established after PE, an accurate biomarker stratifying this would be desirable. Comparably, drawing definitive conclusions about albuminuria use in PE prediction is problematic due to difficult data interpretation. Reasons for this include crude cut-offs in 24 h urinary albumin excretion that distinguish microalbuminuria from macroalbuminuria and the variety of definitions used for both across the studies identified. Better alternatives might be necessary to reflect kidney damage in PE. One alternative discussed is uNGALcc (Kelly et al., 2018), presenting a real-time indicator of pre-albuminuria kidney damage.

The role of insulin resistance and metabolic syndrome in the pathogenesis of PE among women with T1DM is another largely unexplored area, yet several biomarkers discussed in this review can be directly correlated with these states. Increased insulin resistance is a feature of normal pregnancy (Salzer et al., 2015), but excessive resistance has been linked to PE in both the general population (Wolf et al., 2002) and women with T1DM (Gutaj et al., 2015). The importance of insulin resistance in T1DM is becoming increasingly recognized (Kilpatrick et al., 2007), yet few studies have explored its role in PE pathogenesis in this group (Wender-Ozegowska et al., 2011). Adipokines such as leptin, adiponectin and FABP4, are released from metabolically active adipose tissue providing an indirect assessment of the degree of insulin resistance (Gutaj et al., 2015) and upregulation of these molecules has already been associated with PE in the general population (Haugen et al., 2006). Kelly et al. (2017) and Wotherspoon et al. (2016b) are the first to demonstrate a PE biomarker potential for adipokines among women with T1DM. Notably, as Kelly et al. (2017) recommended using a different adipokine for each trimester, the clinical implementation of these biomarkers might be logistically complex. Lipids, dysregulation of which is a known component of the metabolic syndrome together with insulin resistance (Kilpatrick et al., 2007), were also discussed in this review (Kilpatrick et al., 2007). There is uncertainy around the significance of abnormal lipid metabolism in women with T1DM who go on to develop PE, reflected in the inconsistent evidence seen within the current review and Wotherspoon et al. (2016a). Recommendations for use of atherogenic lipid profiles for PE prediction among women with T1DM by some authors contrasted with others finding no differences in triglyceride, HDL-c and LDL-c levels between hypertensive and normotensive groups. Interestingly, the recent findings of Kelly et al. (2019) might explain such conflicting results with different haptoglobin phenotypes. Although contradicting previous studies that found no correlation (Weissgerber et al., 2013), Kelly et al. (2019) were able to showed a significant association between atherogenic dyslipidemia, haptoglobin phenotype 2-2 and PE among women with T1DM. Further investigation is warranted to validate these findings, and of the possibility of haptoglobin phenotype-specific PE biomarkers. Importantly, Hp 2-2 represents only half of the Caucasian T1DM population (Langlois and Delanghe, 1996; Kelly et al., 2019) with further variation likely among other ethnicities.

The newly significant association between rising vitamin D levels and PE in women with pre-existing T1DM was a major finding in this review, in contrast to what has been described previously among women with T1DM (Azar et al., 2011; Vestgaard et al., 2017b) and the evidence for low vitamin D levels among women with PE in the general population (Poniedziałek-Czajkowska and Mierzyński, 2021). This correlation was uncovered due to the different approach adopted by Kelly et al. (2020), measuring both active and precursor vitamin D forms in contrast to their predecessors, who only measured the active form (Azar et al., 2011; Vestgaard et al., 2017b). The findings of Kelly et al. (2020) suggest that other previously non-significant biomarker data could assume significance after methodological re-evaluation. It should be noted that the results of this study were limited by the small number of women included (*n* = 47) and the pathophysiological relevance of the elevated 1,25(OH)_2_D/VDBP ratio in women with T1DM with increased PE risk remains unclear. One explanation could be a compensatory increase of an antioxidant to combat the oxidative stress associated with placental and vascular dysfunction in PE (Kelly et al., 2020). The role of vitamin D supplementation in PE prevention is also notoriously inconclusive in the general population (Poniedziałek-Czajkowska and Mierzyński, 2021).

### Placenta Derived Biomarkers

The lack of association between PAPP-A and PE in women with T1DM (Kapustin et al., 2020) was a notable negative finding in this review. Importantly, as Kapustin et al. (2020) measured PAPP-A at 11−13 + 6 weeks, similar to PAPP-A measurements in studies using the FMF (Thilaganathan and Kalafat, 2019), these results cannot be explained by differences in measurement timeframes. Given the key role of PAPP-A in the first trimester combined PE screening algorithm (Chaemsaithong et al., 2020), prediction models performing well in the general population might not predict PE as accurately among women with T1DM (Guy et al., 2020; Serra et al., 2020). This demonstrates a need to validate general population biomarkers in women with T1DM. The lack of such validation was previously noted by Wotherspoon et al. (2016a). Similarly, placental angiogenic markers were some of the poorest PE predictors discussed in this review (Wotherspoon et al., 2016a; Kelly et al., 2019). Despite the initial optimism surrounding angiogenic factor discovery (Levine et al., 2004), their accuracy has been called into question in the general population (Kleinrouweler et al., 2012), and a recent study of pregnant women with diabetes found that the sFlt-1/PlGF ratio was driven by PlGF in these pregnancies with little difference in sFlt-1 between the PE and normotensive groups (Zen et al., 2019). Therefore, only PlGF might hold a benefit for PE prediction in a system with T1DM.

### Future Directions

Several knowledge gaps were identified that could be addressed by ongoing research efforts. First, the incomplete understanding of PE pathogenesis in women with T1DM is a research priority, as this continues to hinder the prediction and prevention efforts of this disease. The relative trend of maternally-derived PE biomarkers performing better among studies in this review could be a clue to the maternal origins of PE among women with T1DM, worthy of investigation. Moreover, the consistently elevated sEng in pregnant women with T1DM was an intriguing finding (Kelly et al., 2019) and could be an indicator of the maternal cardiovascular preponderance towards PE in the form of pre-existing systemic endothelial dysfunction with long-term T1DM. Elucidating reasons for differences in biomarker performance between groups of women with and without T1DM could also suggest pathophysiological pathways to target in studies investigating preventative approaches for PE beyond aspirin. No clinical trials have been carried out as of yet randomizing women with T1DM to aspirin or placebo.

Second, there was a notable lack of HbA1c studies considering a hypoglycemia risk assessment. Lower HbA1c levels at periconception are correlated with fewer subsequent hypoglycemia episodes (Garey et al., 2020) and Perea et al. (2019) observed a relationship between impaired hypoglycemia awareness, atherogenic dyslipidemia and PE, meriting further investigation. Another largely unexplored area relates to the relationship between PE and diabetic retinopathy. In their meta-analysis, Xiang et al. (2018) showed that presence of diabetic retinopathy increased the risk of PE, however, little other literature exists to explain why and whether there are any modalities reflecting diabetic retinopathy that could be used to predict PE. Indeed, considering that progression of diabetic retinopathy is an important pregnancy consideration in all women with T1DM (Rosenn et al., 1992) and the common pathological considerations between PE, diabetic retinopathy, and nephropathy in the maternal vasculature, this relationship could be important. Regarding methodology, an over-representation of Caucasian women was revealed among the included articles. As this could reduce the external validity of biomarker performance, studies testing their accuracy in other demographics should follow.

### Strengths and Limitations

A particular strength of this review was its robust search strategy. Database searches were supplemented by hand-searching the references of included articles, minimizing the number of unidentified records. Defined inclusion and exclusion criteria were used (Supplementary Appendix S1), and 18 studies were reviewed in total, providing a detailed summary of the current state of research on T1DM-specific PE biomarkers. The novelty of the data was verified by comparing similarities of included studies between this review and Wotherspoon et al. (2016a) (Supplementary Appendix S2). The inability to carry out a meta-analysis in this study was a limitation, attributable to biomarker heterogeneity. We also acknowledge that the included studies were not assessed for risk of bias. Additionally, it was noted that there was some overlap between patient cohorts used by some studies, however, as different biomarkers were investigated in each study, these were included as individual records. Finally, the electronic search was restricted to articles in English only.

## Conclusion

The growing literature on PE biomarkers for women with T1DM has yielded exciting findings in recent years. This narrative review has demonstrated that maternally derived PE biomarkers reflecting glycemic control, insulin resistance and renal dysfunction might be better predictors of PE development among women with T1DM than placental biomarkers. Maternally derived biomarkers could be trialled in with current PE prediction models in the general population to devise an algorithm tailored to PE pathophysiology among women with T1DM. A further investigation of the maternal origins of PE in women with T1DM and reasons for differing biomarker performance might lead novel discoveries in this field.

## Data Availability

The original contributions presented in the study are included in the article/Supplementary Material, further inquiries can be directed to the corresponding author.

## References

[B1] Agrawal S. Cerdeira A. S. Redman C. Vatish M. (2017). Meta-Analysis and Systematic Review to Assess the Role of Soluble FMS-like Tyrosine Kinase-1 and Placenta Growth Factor Ratio in Prediction of Preeclampsia: The SaPPPhirE Study. Hypertension 71 (2), 306–316. 10.1161/HYPERTENSIONAHA.117.10182 29229743

[B2] American College of Obstetrics & Gynaecology (ACOG) (2018). ACOG Practice Bulletin No. 201: Pregestational Diabetes Mellitus. Obstet. Gynecol. 132 (6), e228–e248. 10.1097/aog.0000000000002960 30461693

[B3] Azar M. Basu A. Jenkins A. J. Nankervis A. J. Hanssen K. F. Scholz H. (2011). Serum Carotenoids and Fat-Soluble Vitamins in Women with Type 1 Diabetes and Preeclampsia. Diabetes Care 34 (6), 1258–1264. 10.2337/dc10-2145 21498785 PMC3114346

[B4] Azzoug S. Chentli F. (2016). Microangiopathy and Pregnancy. J. Pak Med. Assoc. 66 (9 Suppl. 1), S52–S55. 27582154

[B5] Basu A. Yu J. Y. Jenkins A. J. Nankervis A. J. Hanssen K. F. Henriksen T. (2015). Trace Elements as Predictors of Preeclampsia in Type 1 Diabetic Pregnancy. Nutr. Res. 35 (5), 421–430. 10.1016/j.nutres.2015.04.004 25912764 PMC4442033

[B6] Brownlee M. (2001). Biochemistry and Molecular Cell Biology of Diabetic Complications. Nature 414 (6865), 813–820. 10.1038/414813a 11742414

[B7] Caritis S. Sibai B. Hauth J. Lindheimer M. D. Klebanoff M. Thom E. (1998). Low-Dose Aspirin to Prevent Preeclampsia in Women at High Risk. N. Engl. J. Med. 338 (11), 701–705. 10.1056/nejm199803123381101 9494145

[B8] Castiglioni M. T. Valsecchi L. Cavoretto P. Pirola S. Di Piazza L. Maggio L. (2014). The Risk of Preeclampsia beyond the First Pregnancy Among Women with Type 1 Diabetes Parity and Preeclampsia in Type 1 Diabetes. Pregnancy Hypertens. Int. J. Women's Cardiovasc. Health 4 (1), 34–40. 10.1016/j.preghy.2013.09.001 26104252

[B9] Cavero-Redondo I. Martínez-Vizcaíno V. Soriano-Cano A. Martínez-Hortelano J. A. Sanabria-Martínez G. Álvarez-Bueno C. (2018). Glycated Haemoglobin A1c as a Predictor of Preeclampsia in Type 1 Diabetic Pregnant Women: A Systematic Review and Meta-Analysis. Pregnancy Hypertens. 14, 49–54. 10.1016/j.preghy.2018.04.004 30527118

[B10] Chaemsaithong P. Sahota D. Poon L. C. (2020). First Trimester Preeclampsia Screening and Prediction. Am. J. Obstet. Gynecol. 226, S1071. 10.1016/j.ajog.2020.07.020 32682859

[B11] Ekbom P. Damm P. Feldt-Rasmussen B. Feldt-Rasmussen U. Mølvig J. Mathiesen E. R. (2001). Pregnancy Outcome in Type 1 Diabetic Women with Microalbuminuria. Diabetes Care 24 (10), 1739–1744. 10.2337/diacare.24.10.1739 11574435

[B12] Finnegan C. Dicker P. Fernandez E. Tully E. Higgins M. Daly S. (2019). Investigating the Role of Early Low-Dose Aspirin in Diabetes: A Phase III Multicentre Double-Blinded Placebo-Controlled Randomised Trial of Aspirin Therapy Initiated in the First Trimester of Diabetes Pregnancy. Contemp. Clin. Trials Commun. 16, 100465. 10.1016/j.conctc.2019.100465 31701039 PMC6831706

[B13] Garey C. Lynn J. Floreen Sabino A. Hughes A. McAuliffe-Fogarty A. (2020). Preeclampsia and Other Pregnancy Outcomes in Nulliparous Women with Type 1 Diabetes: a Retrospective Survey. Gynecol. Endocrinol. 36, 982–985. 10.1080/09513590.2020.1749998 32281439

[B14] Gordin D. Hiilesmaa V. Fagerudd J. Rönnback M. Forsblom C. Kaaja R. (2007). Pre-eclampsia but Not Pregnancy-Induced Hypertension Is a Risk Factor for Diabetic Nephropathy in Type 1 Diabetic Women. Diabetologia 50 (3), 516–522. 10.1007/s00125-006-0544-5 17216281

[B15] Gordin D. Kaaja R. Forsblom C. Hiilesmaa V. Teramo K. Groop P.-H. (2013). Pre-eclampsia and Pregnancy-Induced Hypertension Are Associated with Severe Diabetic Retinopathy in Type 1 Diabetes Later in Life. Acta Diabetol. 50 (5), 781–787. 10.1007/s00592-012-0415-0 22955518

[B16] Gorst C. Kwok C. S. Aslam S. Buchan I. Kontopantelis E. Myint P. K. (2015). Long-term Glycemic Variability and Risk of Adverse Outcomes: A Systematic Review and Meta-Analysis. Diabetes Care 38 (12), 2354–2369. 10.2337/dc15-1188 26604281

[B17] Gutaj P. Sawicka-Gutaj N. Brązert M. Wender-Ożegowska E. (2015). Insulin Resistance in Pregnancy Complicated by Type 1 Diabetes Mellitus. Do We Know Enough? Ginekol. Pol. 86 (3), 219–223. 10.17772/gp/2065 25920313

[B18] Gutaj P. Zawiejska A. Mantaj U. Wender-Ożegowska E. (2017). Determinants of Preeclampsia in Women with Type 1 Diabetes. Acta Diabetol. 54 (12), 1115–1121. 10.1007/s00592-017-1053-3 28975446 PMC5680366

[B19] Guy G. P. Leslie K. Diaz Gomez D. Forenc K. Buck E. Khalil A. (2020). Implementation of Routine First Trimester Combined Screening for Pre-eclampsia: a Clinical Effectiveness Study. Bjog 128, 149. 10.1111/1471-0528.16361 32613730

[B20] Haugen F. Ranheim T. Harsem N. K. Lips E. Staff A. C. Drevon C. A. (2006). Increased Plasma Levels of Adipokines in Preeclampsia: Relationship to Placenta and Adipose Tissue Gene Expression. Am. J. Physiology-Endocrinology Metabolism 290 (2), E326–E333. 10.1152/ajpendo.00020.2005 16144822

[B21] Hsu C. D. Tan H. Y. Hong S. F. Nickless N. A. Copel J. A. (1996). Strategies for Reducing the Frequency of Preeclampsia in Pregnancies with Insulin-dependent Diabetes Mellitus. Am. J. Perinatol. 13 (5), 265–268. 10.1055/s-2007-994340 8863944

[B22] Inadera H. Tachibana S. Takasaki I. Tatematsu M. Shimomura A. (2010). Hyperglycemia Perturbs Biochemical Networks in Human Trophoblast BeWo Cells. Endocr. J. 57 (7), 567–577. 10.1507/endocrj.k10e-045 20467164

[B23] Inkster M. E. Fahey T. P. Donnan P. T. Leese G. P. Mires G. J. Murphy D. J. (2006). Poor Glycated Haemoglobin Control and Adverse Pregnancy Outcomes in Type 1 and Type 2 Diabetes Mellitus: Systematic Review of Observational Studies. BMC Pregnancy Childbirth 6, 30. 10.1186/1471-2393-6-30 17074087 PMC1635059

[B24] Kapustin R. V. Kascheeva T. K. Alekseenkova E. N. Shelaeva E. V. (2020). Are the First-Trimester Levels of PAPP-A and Fb-hCG Predictors for Obstetrical Complications in Diabetic Pregnancy? J. Maternal Fetal Neonatal Med. 35, 1113. 10.1080/14767058.2020.1743658 32228094

[B25] Kelly C. B. Wagner C. L. Shary J. R. Leyva M. J. Yu J. Y. Jenkins A. J. (2020). Vitamin D Metabolites and Binding Protein Predict Preeclampsia in Women with Type 1 Diabetes. Nutrients 12 (7), 2048. 10.3390/nu12072048 PMC7400952 32664257

[B26] Kelly C. B. Hookham M. B. Yu J. Y. Jenkins A. J. Nankervis A. J. Hanssen K. F. (2018). Subclinical First Trimester Renal Abnormalities Are Associated with Preeclampsia in Normoalbuminuric Women with Type 1 Diabetes. Diabetes Care 41 (1), 120–127. 10.2337/dc17-1635 29122892 PMC5741157

[B27] Kelly C. B. Hookham M. B. Yu J. Y. Lockhart S. M. Du M. Jenkins A. J. (2017). Circulating Adipokines Are Associated with Pre-eclampsia in Women with Type 1 Diabetes. Diabetologia 60 (12), 2514–2524. 10.1007/s00125-017-4415-z 28875223 PMC9597852

[B28] Kelly C. B. Yu J. Y. Jenkins A. J. Nankervis A. J. Hanssen K. F. Garg S. K. (2019). Haptoglobin Phenotype Modulates Lipoprotein-Associated Risk for Preeclampsia in Women with Type 1 Diabetes. J. Clin. Endocrinol. Metab. 104 (10), 4743–4755. 10.1210/jc.2019-00723 31219590

[B29] Kilpatrick E. S. Rigby A. S. Atkin S. L. (2007). Insulin Resistance, the Metabolic Syndrome, and Complication Risk in Type 1 Diabetes. Diabetes Care 30 (3), 707–712. 10.2337/dc06-1982 17327345

[B30] Kleinrouweler C. Wiegerinck M. Ris-Stalpers C. Bossuyt P. van der Post J. von Dadelszen P. (2012). Accuracy of Circulating Placental Growth Factor, Vascular Endothelial Growth Factor, Soluble Fms-like Tyrosine Kinase 1 and Soluble Endoglin in the Prediction of Pre-eclampsia: a Systematic Review and Meta-Analysis. Bjog 119 (7), 778–787. 10.1111/j.1471-0528.2012.03311.x 22433027

[B31] Klemetti M. M. Laivuori H. Tikkanen M. Nuutila M. Hiilesmaa V. Teramo K. (2016). White's Classification and Pregnancy Outcome in Women with Type 1 Diabetes: a Population-Based Cohort Study. Diabetologia 59 (1), 92–100. 10.1007/s00125-015-3787-1 26474777

[B32] Langlois M. R. Delanghe J. R. (1996). Biological and Clinical Significance of Haptoglobin Polymorphism in Humans. Clin. Chem. 42 (10), 1589–1600. 10.1093/clinchem/42.10.1589 8855140

[B33] Lauszus F. F. Fuglsang J. (2016). IGF-1 Is Associated with Fetal Growth and Preterm Delivery in Type 1 Diabetic Pregnancy. Gynecol. Endocrinol. 32 (6), 488–491. 10.3109/09513590.2015.1134477 26758936

[B34] Leguizamón G. Trigubo D. Pereira J. I. Vera M. F. Fernández J. A. (2015). Vascular Complications in the Diabetic Pregnancy. Curr. Diab Rep. 15 (4), 22. 10.1007/s11892-015-0586-5 25732848

[B35] Levine R. J. Maynard S. E. Qian C. Lim K.-H. England L. J. Yu K. F. (2004). Circulating Angiogenic Factors and the Risk of Preeclampsia. N. Engl. J. Med. 350 (7), 672–683. 10.1056/nejmoa031884 14764923

[B36] Lövestam-Adrian M. Agardh C.-D. Åberg A. Agardh E. (1997). Pre-eclampsia Is a Potent Risk Factor for Deterioration of Retinopathy during Pregnancy in Type 1 Diabetic Patients. Diabet. Med. 14 (12), 1059–1065. 10.1002/(sici)1096-9136(199712)14:12<1059::aid-dia505>3.0.co;2-8 9455934

[B37] Maresh M. J. A. Holmes V. A. Patterson C. C. Young I. S. Pearson D. W. M. Walker J. D. (2015). Glycemic Targets in the Second and Third Trimester of Pregnancy for Women with Type 1 Diabetes. Diabetes Care 38 (1), 34–42. 10.2337/dc14-1755 25368104

[B38] Mathiesen E. R. (2016). Pregnancy Outcomes in Women with Diabetes-Lessons Learned from Clinical Research: The 2015 Norbert Freinkel Award Lecture. Diabetes Care 39 (12), 2111–2117. 10.2337/dc16-1647 27879355

[B39] National Institue for Health and Care Excellence (NICE) (2019). Hypertension in Pregnancy: Diagnosis and Management. NICE. 31498578

[B40] National Institute for Health and Care Excellence (NICE) (2015). Diabetes in Pregnancy: Management of Diabetes and its Complications from Preconception to the Postnatal Period. NICE. 25950069

[B41] Nielsen L. H. Jensen B. L. Fuglsang J. Andersen L. L. T. Jensen D. M. Jørgensen J. S. (2018). Urine Albumin Is a Superior Predictor of Preeclampsia Compared to Urine Plasminogen in Type I Diabetes Patients. J. Am. Soc. Hypertens. 12 (2), 97–107. 10.1016/j.jash.2017.12.003 29305116

[B42] Perea V. Bertran B. Bellart J. Orois A. Giménez M. Conget I. (2019). Impaired Awareness of Hypoglycaemia: A New Risk Factor for Adverse Pregnancy Outcomes in Type 1 Diabetes. Diabetes Metab. Res. Rev. 35 (7), e3176. 10.1002/dmrr.3176 31066196

[B43] Poniedziałek-Czajkowska E. Mierzyński R. (2021). Could Vitamin D Be Effective in Prevention of Preeclampsia? Nutrients 13 (11), 3854. 10.3390/nu13113854 34836111 PMC8621759

[B44] Poon L. C. Y. Kametas N. A. Maiz N. Akolekar R. Nicolaides K. H. (2009). First-Trimester Prediction of Hypertensive Disorders in Pregnancy. Hypertension 53, 812–818. 10.1161/hypertensionaha.108.127977 19273739

[B45] Rolnik D. L. O'Gorman N. Roberge S. Bujold E. Hyett J. Uzan S. (2017). Early Screening and Prevention of Preterm Pre-eclampsia with Aspirin: Time for Clinical Implementation. Ultrasound Obstet. Gynecol. 50 (5), 551–556. 10.1002/uog.18899 28887883

[B46] Rolnik D. L. Wright D. Poon L. C. O’Gorman N. Syngelaki A. de Paco Matallana C. (2017). Aspirin versus Placebo in Pregnancies at High Risk for Preterm Preeclampsia. N. Engl. J. Med. 377, 613–622. 10.1056/nejmoa1704559 28657417

[B47] Rosenn B. Miodovnik M. Kranias G. Khoury J. Combs C. A. Mimouni F. (1992). Progression of Diabetic Retinopathy in Pregnancy: Association with Hypertension in Pregnancy. Am. J. Obstetrics Gynecol. 166 (4), 1214–1218. 10.1016/s0002-9378(11)90608-5 1566772

[B48] Salzer L. Tenenbaum-Gavish K. Hod M. (2015). Metabolic Disorder of Pregnancy (Understanding Pathophysiology of Diabetes and Preeclampsia). Best Pract. Res. Clin. Obstetrics Gynaecol. 29 (3), 328–338. 10.1016/j.bpobgyn.2014.09.008 25481558

[B49] Say L. Chou D. Gemmill A. Tunçalp Ö. Moller A.-B. Daniels J. (2014). Global Causes of Maternal Death: a WHO Systematic Analysis. Lancet Glob. Health 2 (6), e323–e333. 10.1016/s2214-109x(14)70227-x 25103301

[B50] Serra B. Mendoza M. Scazzocchio E. Meler E. Nolla M. Sabrià E. (2020). A New Model for Screening for Early-Onset Preeclampsia. Am. J. Obstet. Gynecol. 222 (6), 608. 10.1016/j.ajog.2020.01.020 31972161

[B51] Thilaganathan B. Kalafat E. (2019). Cardiovascular System in Preeclampsia and beyond. Hypertension 73, 522–531. 10.1161/hypertensionaha.118.11191 30712425 PMC6380450

[B52] Tsiakkas A. Duvdevani N. Wright A. Wright D. Nicolaides K. H. (2015). Serum Placental Growth Factor in the Three Trimesters of Pregnancy: Effects of Maternal Characteristics and Medical History. Ultrasound Obstet. Gynecol. 45 (5), 591–598. 10.1002/uog.14811 25653039

[B53] Vestgaard M. Secher A. L. Ringholm L. Jensen J.-E. B. Damm P. Mathiesen E. R. (2017). Vitamin D Insufficiency, Preterm Delivery and Preeclampsia in Women with Type 1 Diabetes - an Observational Study. Acta Obstet. Gynecol. Scand. 96 (10), 1197–1204. 10.1111/aogs.13180 28590567

[B54] Vestgaard M. Sommer M. C. Ringholm L. Damm P. Mathiesen E. R. (2017). Prediction of Preeclampsia in Type 1 Diabetes in Early Pregnancy by Clinical Predictors: a Systematic Review. J. Maternal-Fetal Neonatal Med. 31 (14), 1933–1939. 10.1080/14767058.2017.1331429 28574296

[B55] Villa P. Kajantie E. Räikkönen K. Pesonen A.-K. Hämäläinen E. Vainio M. (2013). Aspirin in the Prevention of Pre-eclampsia in High-Risk Women: a Randomised Placebo-Controlled PREDO Trial and a Meta-Analysis of Randomised Trials. Bjog 120 (1), 64–74. 10.1111/j.1471-0528.2012.03493.x 23126307

[B56] Weissgerber T. Gandley R. Roberts J. Patterson C. Holmes V. Young I. (2013). Haptoglobin Phenotype, Pre-eclampsia, and Response to Supplementation with Vitamins C and E in Pregnant Women with Type-1 Diabetes. Bjog 120 (10), 1192–1199. 10.1111/1471-0528.12288 23718253 PMC3860176

[B57] Weissgerber T. L. Mudd L. M. (2015). Preeclampsia and Diabetes. Curr. Diab Rep. 15 (3), 9. 10.1007/s11892-015-0579-4 25644816 PMC4317712

[B58] Wender-Ozegowska E. Zawiejska A. Michalowska-Wender G. Iciek R. Wender M. Brazert J. (2011). Metabolic Syndrome in Type 1 Diabetes Mellitus. Does it Have Any Impact on the Course of Pregnancy? J. Physiol. Pharmacol. 62 (5), 567–573. 22204805

[B59] White P. (1949). Pregnancy Complicating Diabetes. Am. J. Med. 7 (5), 609–616. 10.1016/0002-9343(49)90382-4 15396063

[B60] Wolf M. Sandler L. Muñoz K. Hsu K. Ecker J. L. Thadhani R. (2002). First Trimester Insulin Resistance and Subsequent Preeclampsia: a Prospective Study. J. Clin. Endocrinol. Metab. 87 (4), 1563–1568. 10.1210/jcem.87.4.8405 11932283

[B61] Wotherspoon A. C. Holmes V. A. Patterson C. C. Young I. S. McCance D. (2016). Serum FABP4 Predicts Preeclampsia in Pregnant Women with Type 1 Diabetes. Diabetes 65 (Suppl. 1), A352. 10.2337/dc16-0803

[B62] Wotherspoon A. C. Young I. S. McCance D. R. Holmes V. A. (2016). Evaluation of Biomarkers for the Prediction of Pre-eclampsia in Women with Type 1 Diabetes Mellitus: A Systematic Review. J. Diabetes its Complicat. 30 (5), 958–966. 10.1016/j.jdiacomp.2016.02.001 26900097

[B63] Xiang L.-J. Wang Y. Lu G.-Y. Huang Q. (2018). Association of the Presence of Microangiopathy with Adverse Pregnancy Outcome in Type 1 Diabetes: A Meta-Analysis. Taiwan. J. Obstetrics Gynecol. 57 (5), 659–664. 10.1016/j.tjog.2018.08.008 30342646

[B64] Zen M. Padmanabhan S. Zhang K. Kirby A. Cheung N. W. Lee V. W. (2019). Urinary and Serum Angiogenic Markers in Women with Preexisting Diabetes during Pregnancy and Their Role in Preeclampsia Prediction. Diabetes Care 43 (1), 67–73. 10.2337/dc19-0967 31601637

