# Supplementary material for: Pre-Eclampsia Biomarkers for Women With Type 1 Diabetes Mellitus: A Comprehensive Review of Recent Literature
Source: Front Bioeng Biotechnol. 2022 May 26;10:809528. doi: 10.3389/fbioe.2022.809528 (PMC9198830; doi:10.3389/fbioe.2022.809528)
Supplement: Supplementary file 1 [file Table1.DOCX]

# APPENDIX I

**Table 4**

Inclusion and exclusion criteria

| Inclusion criteria | Exclusion criteria |
| --- | --- |
| - Biomarker measured in the plasma, serum or urine of women with T1DM - Biomarker levels were correlated with PE development - Baseline characteristics of study participants were accessible for original articles - Singleton pregnancies | - PE was not reported or reported as part of a composite outcome - Type of diabetes was not well defined - Pregnancies not complicated by diabetes - Pregnancies with comorbidities other than T1DM or T1DM-related complications - Multiple pregnancy - No baseline characteristics of participants reported in original studies - Biomarkers were not measured in plasma, serum or urine - Biomarkers were self-reported by participants - Studies focusing on diagnosis or therapeutics - Inappropriate study format |

# APPENDIX II

An assessment of overlapping data was made as part of analysis of the systematic reviews included in this review (**Table 5**). The four studies examining HbA1c included in Wotherspoon et al.^16^ were also used in Cavero-Redondo et al.^17^ with the addition of another study. Cavero-Redondo et al.^17^ was included due the new insights provided by a meta-analysis. Only one HbA1c study was duplicated between Vestgaard et al.^18^and Cavero-Redondo et al.^17^. Only two out of eight microalbuminuria studies across the selection of Wotherspoon et al.^16^, Vestgaard et al.^18^ and Xiang et al.^19^ overlapped. Hence, the conclusions regarding microalbuminuria in the latter two systematic reviews were considered. Additionally, two studies included in this review as distinct records were also included by systematic reviews - Gutaj et al.^21^ was included in the Xiang et al.^19^ meta-analysis and Klemetti et al.^28^ was included by Vestgaard et al.^18^ and Xiang et al.^19^. Overlap in reporting results was avoided, though, as Xiang et al.^19^ used the data from Gutaj et al.^21^ in a combined “vasculopathy group” which was not considered here, and data originating from Klemetti et al.^28^ in the Vestgaard et al.^18^ systematic review was not included here.

**Table 5**

Analysis of overlapping evidence within the systematic reviews of PE biomarkers in women with T1DM

| Study | HbA1c | Microalbuminuria | Macroalbuminuria |
| --- | --- | --- | --- |
| Wotherspoon 2016^16^ | Holmes *et al*., (2011)  Hiilesmaa *et al*., (2000)  Jensen *et al*., (2010)  Temple *et al*., (2006) | Ekbom *et al*., (1999)  Ekbom *et al*., (2000)  Jensen *et al*., (2010) | N/A |
| Vestgaard 2017^18^ | Temple *et al*., (2006)  Castiglioni *et al*., (2014)  Hanson *et al*., (1998)  Klemetti *et al*., (2016)  Hsu *et al*., (1996)  Ekbom *et al* 2001 | Castiglioni *et al*., (2014)  Ekbom *et al*., (2001)  Lauszus *et al*., (2001) | Howarth *et al*., (2007)  Ekbom *et al*., (2001)  Lauszus *et al*., (2001)  Hanson & Persson *et al*., (1998)  Klemetti *et al*., (2016) |
| Cavero-Redondo 2018^17^ | Hiilesmaa *et al*., (2000)  Holmes *et al*., (2011)  Jensen *et al*., (2010)  Temple *et al*., (2006)  Todorova *et al*., (2007) | N/A | N/A |
| Xiang 2018^19^ | N/A | Castiglioni *et al*., (2014)  de Oliveira Gomes *et al*., (2016)  Jensen *et al*., (2010)  Nielsen *et al*., (2009) | Haeri *et al*., (2008)  Hanson & Persson *et al*., (1998)  Howarth *et al*., (2007)  Klemetti *et al*., (2016)  Nielsen *et al*., (2009) |

Underlined – overlapping studies; Underlined – studies included as a separate article in the current review.
